# The Application of Biopsy Density in Transperineal Templated-Guided Biopsy Patients With PI-RADS<3

**DOI:** 10.3389/fonc.2022.918300

**Published:** 2022-06-08

**Authors:** Hai Zhu, Xue-fei Ding, Sheng-ming Lu, Ning Ding, Shi-yi Pi, Zhen Liu, Qin Xiao, Liang-yong Zhu, Yang Luan, Yue-xing Han, Hao-peng Chen, Zhong Liu

**Affiliations:** ^1^ Department of Urology, Northern Jiangsu People’s Hospital, Yangzhou, China; ^2^ Graduate School, Dalian Medical University, Dalian, China; ^3^ Biobank, Northern Jiangsu People’s Hospital, Yangzhou, China; ^4^ Operating Department, Northern Jiangsu People’s Hospital, Yangzhou, China; ^5^ Pathology Department, Northern Jiangsu People’s Hospital, Yangzhou, China; ^6^ Clinical Medical College, Yangzhou University, Yangzhou, China

**Keywords:** prostate cancer, clinically significant prostate cancer, PI-RADS, mpMRI, transperineal template-guided prostate biopsy

## Abstract

**Background:**

In patients with multiparameter magnetic resonance imaging (mpMRI) low-possibility but highly clinical suspicion of prostate cancer, the biopsy core is unclear. Our study aims to introduce the biopsy density (BD; the ratio of biopsy cores to prostate volume) and investigates the BD-predictive value of prostate cancer and clinically significant prostate cancer (csPCa) in PI-RADS<3 patients.

**Methods:**

Patients underwent transperineal template–guided prostate biopsy from 2012 to 2022. The inclusion criteria were PI-RADS<3 with a positive digital rectal examination or persistent PSA abnormalities. BD was defined as the ratio of the biopsy core to the prostate volume. Clinical data were collected, and we grouped the patients according to pathology results. Kruskal–Wallis test and chi-square test were used in measurement and enumeration data, respectively. Logistics regression was used to choose the factor associated with positive biospy and csPCa. The receiver operating characteristic (ROC) curve was used to evaluate the ability to predict csPCa.

**Results:**

A total of 115 patients were included in our study. Biopsy was positive in 14 of 115 and the International Society of Urological Pathology grade groups 2–5 were in 7 of all the PCa patients. The BD was 0.38 (0.24-0.63) needles per milliliter. Binary logistics analysis suggested that PSAD and BD were correlated with positive biopsy. Meanwhile, BD and PSAD were associated with csPCa. The ROC curve illustrated that BD was a good parameter to predict csPCa (AUC=0.80, 95% CI: 0.69-0.91, p<0.05). The biopsy density combined with PSAD increased the prediction of csPCa (AUC=0.90, 95% CI: 0.85-0.97, p<0.05). The cut-off value of the BD was 0.42 according to the Youden index.

**Conclusion:**

In PI-RADS<3 patients, BD and PSAD are related to csPCa. A biopsy density of more than 0.42 needles per millimeter can increase the csPCa detection rate, which should be considered as an alternative biopsy method when we perform prostate biopsy in patients with PI-RADS<3.

## Background

Multiparametric MRI (mpMRI) is an important tool for detecting prostate cancer. With the prostate imaging reporting and data system (PI-RADS) proposed and developed by the European Society of Uroradiology\, this system acts a non-negligible role. However, mpMRI misses 50% of tumor foci (1) and some regions of prostate cancer lesions are invisible in mpMRI (2). For those mpMRI low-possibility but highly clinical suspicion of prostate cancer patients, transperineal template-guided prostate biopsy is necessary.

The biopsy core is an important clinical parameter. Before the PI-RADS was proposed, the Vienna nomogram was constructed to determine the number of biopsy specimens based on the prostate volume and age (3). Nowadays, for those patients with mpMRI low-possibility but highly clinical suspicion of prostate cancer, the biopsy core varies from different institution, and the optimal number of biopsy specimens for PI-RADS<3 patients is unknown. Can the biopsy core be minimized while maximizing the detection rate of prostate cancer? In this paper, we introduce a clinical parameter: biopsy density (BD) (4) (definition: the ratio of the biopsy core to the prostate volume). We tried to optimize the biopsy strategy for PI-RADS<3 patients.

## Materials and Methods

### Study Population

**Figure 1:**
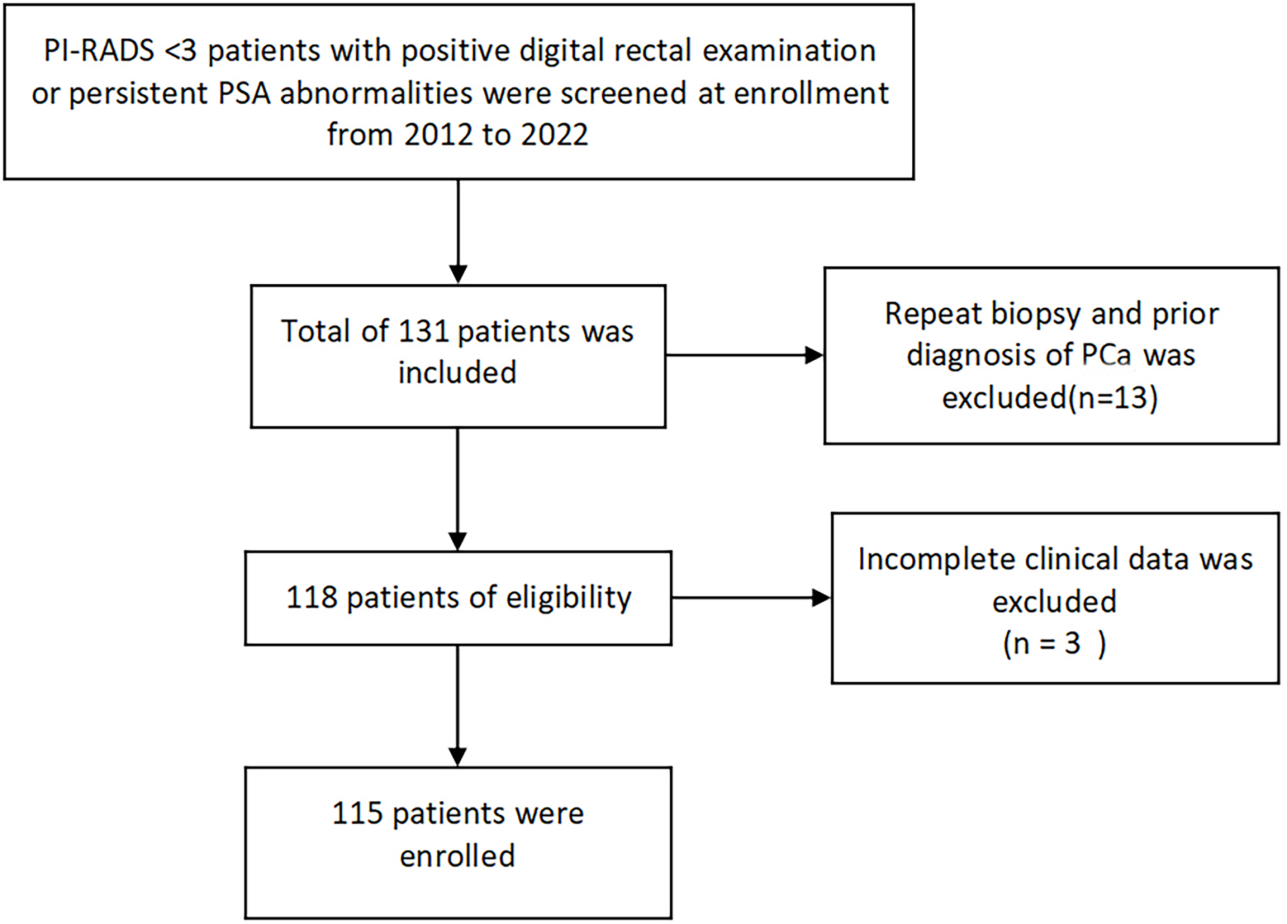
Flowchart of MRI low-possibility patients with positive DRE or persistent PSA >4 ng/ml.

This study is a retrospective analysis of patients who underwent transperineal template-guided prostate biopsy in the Department of Urology, Northern Jiangsu People’s Hospital from May 2012 to April 2022. The inclusion criteria are as follows: 1. patients with PI-RADS<3 and 2. digital rectal examination (DRE) finding nodules or persistent prostate-specific antigen (PSA) >4 ng/ml. Meanwhile, the exclusion criteria are as follows: 1. Patients with prior biopsy; 2. patients with a prior diagnosis of prostate cancer; and 3. patients with incomplete clinical data. Meeting any of the above was excluded (**Figure 1**).

### Method

Before prostate biopsy, DRE and PSA were examined in outpatient service and patients with positive DRE or PSA>4 ng/ml were suggested to perform mpMRI, which was a 3.0-T MR scanner (Signa HDxt; GE Medical Systems, Milwaukee, WI, United States) including T1-weighted images (T1WIs), T2-weighted images (T2WIs), dynamic contrast enhancement (DCE), diffusion-weighted imaging (DWI), and apparent diffusion coefficient (ADC). All mpMRI images were evaluated by one experienced radiologist without the knowledge of patients’ information. The radiologist scored all the patients’ mp-MRI according to PI-RADS v2 (5). For patients with a PI-RADS score 1–2, we performed 14 regions systemic biopsy.

**Figure 2:**
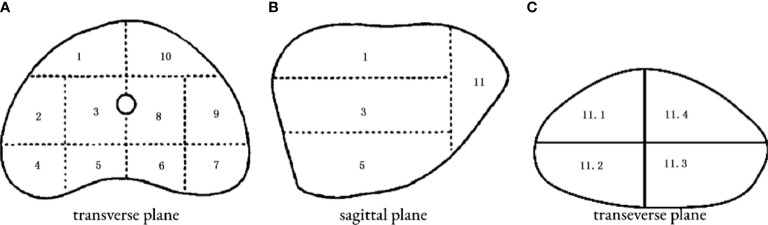
14 regions of transperineal template–guided prostate biopsy. **(A)** Transverse plane of the prostate base. 1: Right anterior region; 2: right lateral middle region; 3: right middle region; 4: right posterior-lateral horn region; 5: right back region; 6: left back region; 7: left posterior lateral horn region; 8: left middle region; 9: left lateral middle region; 10: left anterior region. **(B)** Sagittal plane of the prostate. **(C)** Transverse plane of the prostate apex. 11.1: Right anterior region; 11.2: right back region; 11.3: left back region; 11.4: left anterior region.

The patient was placed in a lithotomy position, and the biplanar TRUS probe (Flex Focus 1202 rectal ultrasound; BK, Naerum, Denmark) was fixed to the stepper and placed into the rectum. According to the anatomical characteristics of the prostate, the prostate was divided into two planes and 14 regions (**Figure 2**). We performed 1–4 cores in every region using an 18 gauge biopsy device (MC1820; BARD, New Jersey NJ, United States) according to the prostate volume under the real-time monitoring of ultrasound.

After prostate biopsy, all pathological sections were reported by the same pathologist of Northern Jiangsu People’s Hospital according to the Gleason score and International Society of Urological Pathology (ISUP) grade: Grade Group (GG) 1 (Gleason score ≤ 6); GG 2 (Gleason score 3 + 4 = 7); GG 3 (Gleason score 4 + 3 = 7); GG 4 (Gleason score 4 + 4 = 8; 3 + 5 = 8; 5 + 3 = 8); and GG 5 (Gleason scores 9-10) (6). Clinically significant prostate cancer was defined as Gleason score 3 + 4 = 7 and higher (≥ISUP GG 2) (7). Simultaneously, biopsy cores were counted in terms of the actual number of tissue specimens obtained at the pathology department of Northern Jiangsu People’s Hospital after the biopsy. The length, width, and height of the prostate were measured strictly according to the method proposed by PI-RADS v2, and the prostate volume was calculated according to the ellipsoidal volume formula: length * width * height * 0.52.

### Statistical Analysis

SPSS (version 19.0; IBM Corp, Armonk, NY, United States) statistical software was used for data analysis. Age, PSA, prostate volume, biopsy cores, PSA density (PSA density, PSAD), BD, and pathology data were collected. Data were described using X ± S if they obeyed a normal distribution and t-test was used; if they did not obey a normal distribution, they were described using M(Q1, Q3) and a nonparametric test was used. All clinical parameters did not conform to the normal distribution, so we used M (Q1, Q3) to describe measurement data. The chi-square test was used for enumeration data. In this study, patients were divided into the negative biopsy group and positive group according to biopsy results; all the patients were classified into the non-clinically significant prostate cancer (non-csPCa) group and csPCa group according to the definition of csPCa in terms of the pathological results. Non-csPCa included biopsy- negative and ISUP GG 1 PCa patients. Binary logistic regression was used to determine the risk factors for Pca and csPCa. The area under the curve of the receiver operating characteristic curve (ROC) was calculated to evaluate the ability of the BD to predict PCa and csPCa. A threshold value for the BD was found using the Youden index. A two-tailed P-value <0.05 was used to indicate statistical significance.

### Result

A total of 115 patients were included in this study. Age was 66(60-73) years, with a PSA of 8.03(5.9-11.83) ng/ml, prostate volume of 56(40-85) ml, biopsy core of 21(15-27), and PSA density (PSAD) of 0.13(0.08-0.25) ng/ml/ml. Biopsy results were positive in 14/115 (12.17%) and ISUP GG 2–5 patients were in 7/115 (6.09%). The BD was 0.38 (0.24-0.63).

**Figure 3:**
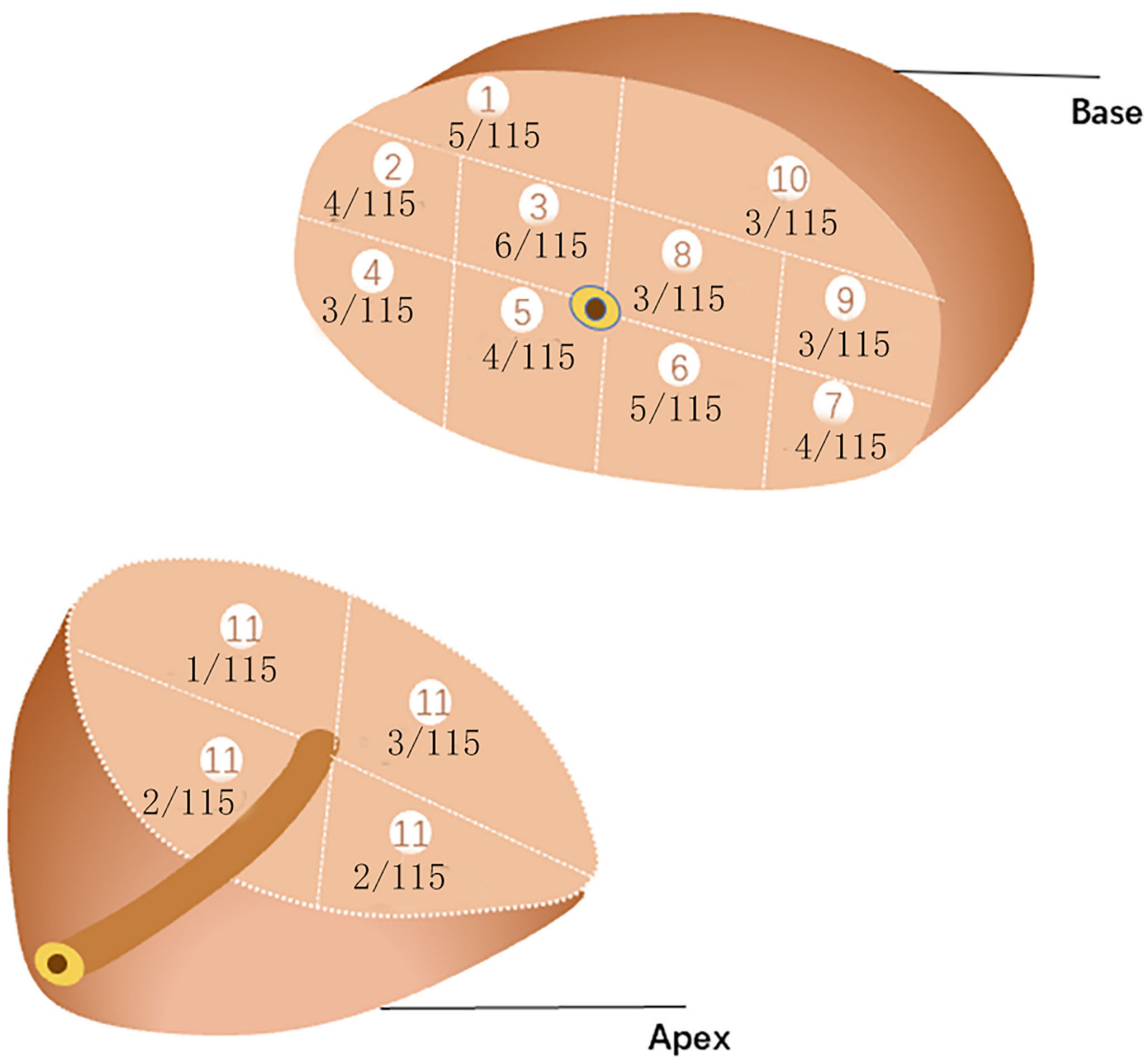
PCa detection rate of each region.

We counted the biopsy cores in each of the 14 regions. The average number in region 1 was 1.5 (1~2) core, 2 (1~2) cores in region 2, 2 (1~2) cores in region 3, 2(1~2) cores in region 4, 2 (1~2) cores in region 5, 2 (1~2) cores in region 6, 2 (1~2) cores region 7, 2 (1~2)cores in region 8, 2 (1~2) cores in region 9, 2 (1~2) cores in region 10, and 2(2~4) cores in region 11 of 4 subregions.The biopsy-positive rates for each region were 4.35% for region 1, 3.45% for region 2, 5.22% for region 3, 2.61% for region 4, 3.45% for region 5, 4.35% for region 6, 3.45% for region 7, 2.61% for region 8, 2.61% for region 9, 2.61% for region 10, and 5.21% for region 11 of 4 subregions (**Figure 3**). The chi-square test suggested that the positive rates of each region were not statistically significant (p>0.05), as well as the csPCa detection rate.

The baseline data of the negative- and positive-biopsy groups and baseline information in the csPCa and non-csPCa groups were as follows (**Tables 1**, **2**). The age, PSA, and biopsy core did not differ between the groups (P > 0.05). Patients in the biopsy-positive group had higher PSAD and BD compared with those in the biopsy-negative group (P < 0.05). Patients in the csPCa group had higher PSAD and BD compared with those in the non-csPCa group (P < 0.05).

**Table 1:** Comparison of baseline information of patients in the biopsy-positive and negative groups.

	Negative Group	Positive Group	P
Age, year	66 (59-73)	64 (62.5-72.5)	0.966
PSA, ng/ml	8.03 (5.88-11.70)	8.9 (5.58-16.92)	0.647
Prostate volume, ml	57 (40.5-89)	44.5 (27.25-59.5)	0.044
Biopsy core, needle	20 (15-26.5)	23.5 (19.5-32.2)	0.06
Biopsy density, needle/ml	0.35 (0.22-0.56)	0.57 (0.46-0.86)	0.004
PSAD, ng/ml/ml	0.13 (0.08-0.24)	0.22 (0.09-0.50)	0.092

**Table 2:** Comparison of baseline information of patients with csPCa and non-csPCa groups.

	Non-csPCa Group	csPCa Group	P
Age, year	66 (59.25-73)	64 (63-68)	0.756
PSA, ng/ml	8.03 (5.90-11.66)	16.7 (4.63-19.41)	0.387
Prostate volume, ml	57 (40-87.75)	41 (25-50)	0.027
Biopsy core, needle	21 (15-27)	21 (16-51)	0.346
Biopsy density, needle/ml	0.36 (0.23-0.56)	0.67 (0.49-1.02)	0.008
PSAD, ng/ml/ml	0.13 (0.08-0.23)	0.41 (0.09-0.73)	0.038

All clinical parameters were first analyzed separately using univariate analysis of logistics, and then statistically significant clinical parameters were included in a binary logistic regression comparing the negative and positive groups separately (**Table 3**). The biopsy density and PSAD were found to be associated with a positive biopsy result. Meanwhile, the BD and PSAD were correlated with csPCa in the non-csPCa and csPCa groups (**Table 4**).

**Table 3:** Results of binary logistic regression analysis (biopsy-negative group vs. biopsy-positive group).

				95% Confidence Interval	
	OR	Wald	P	Lower Bound	Higher Bound
Biopsy density	2.28	4.103	0.043	1.027	5.063
PSAD	3.262	4.011	0.045	1.026	10.378

**Table 4:** Results of binary logistic regression analysis (csPCa group vs. non-csPCa group).

				95% Confidence Interval	
	OR	Wald	P	Lower bound	Higher bound
Biopsy density	3.419	4.875	0.027	1.148	10.184
PSAD	6.798	5.301	0.021	1.33	34.756

**Figure 4:**
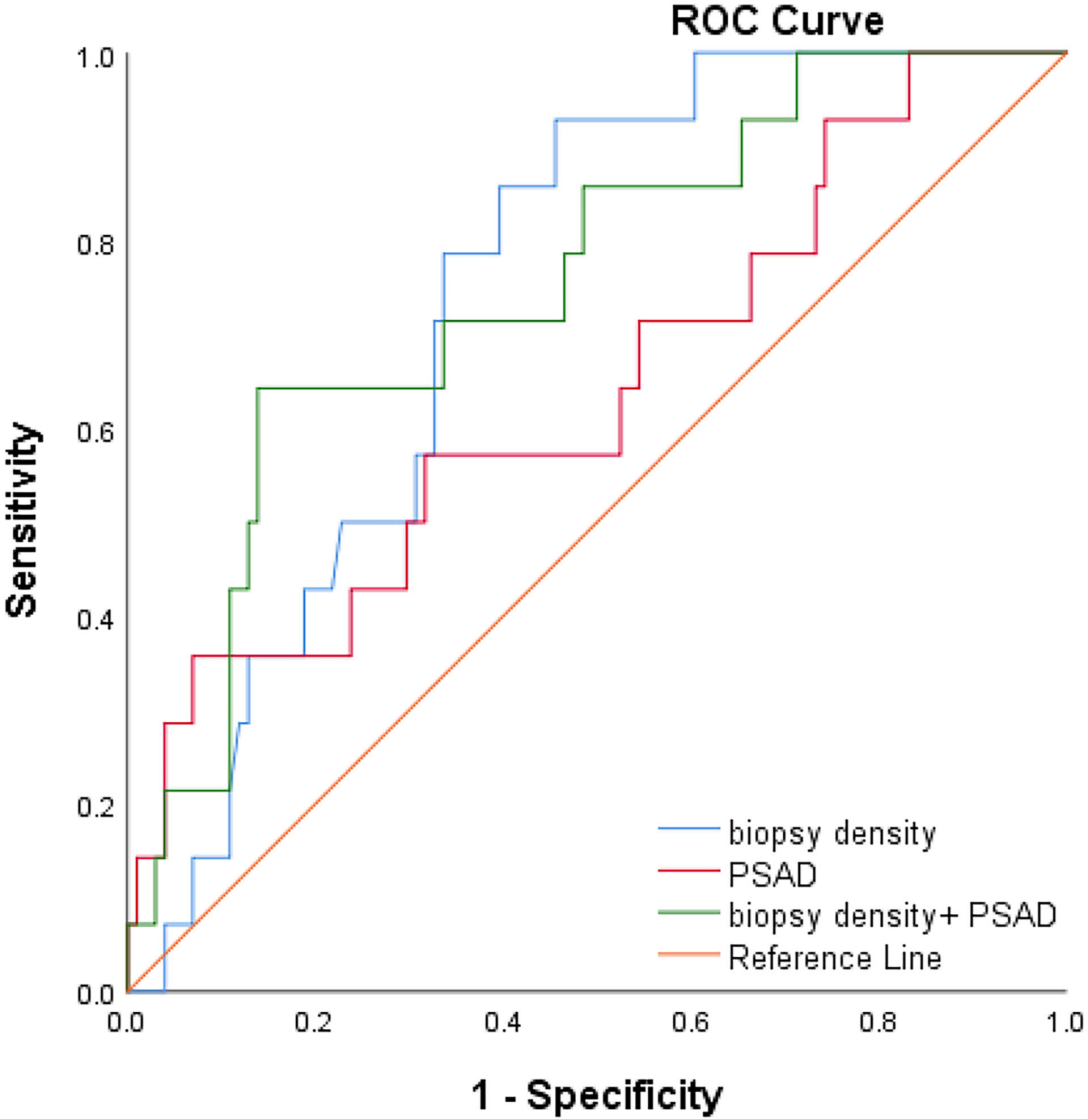
ROC curve of positive biopsy using age, PSAD, BD, and age+BD+PSAD.

**Figure 5:**
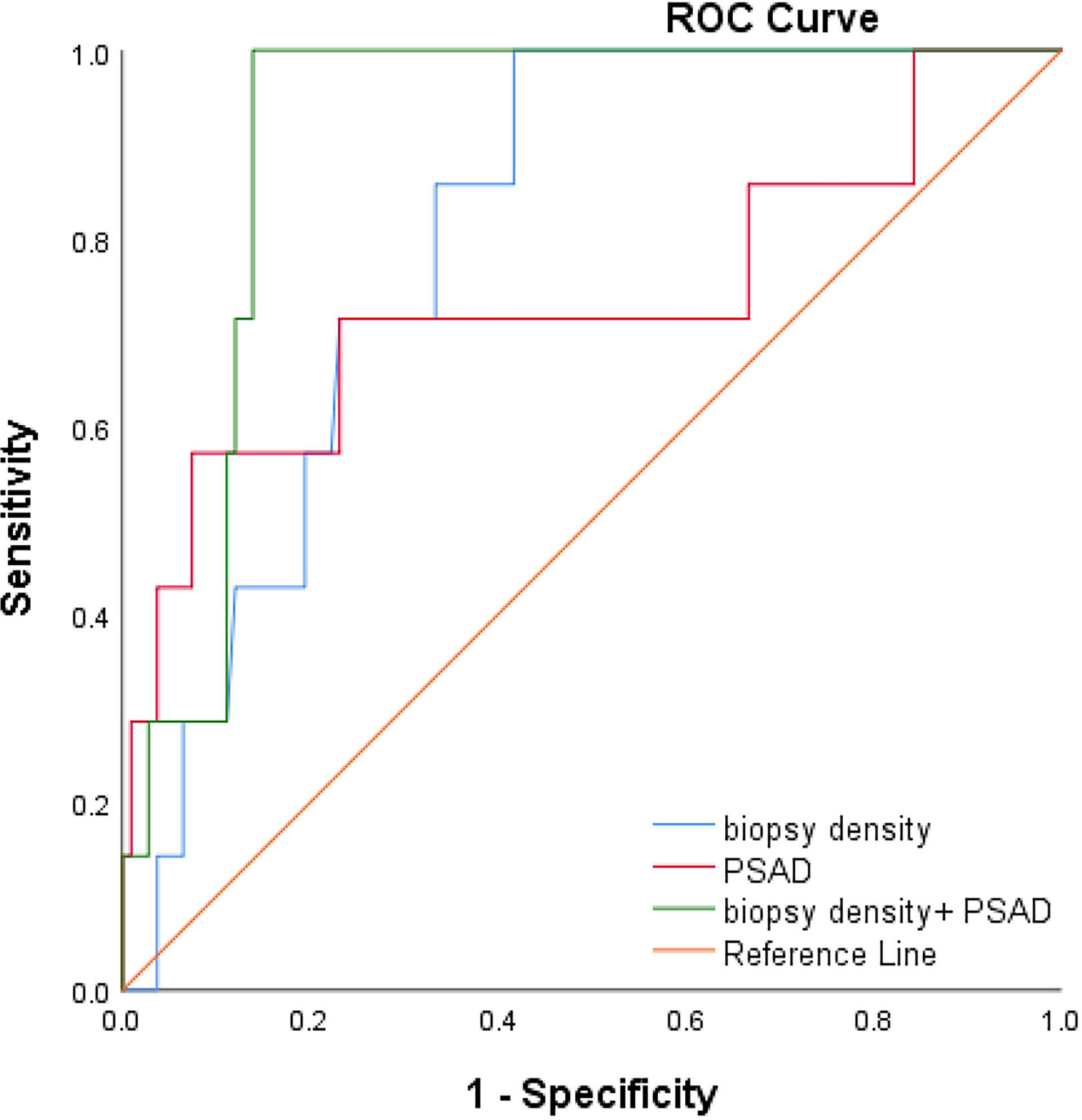
ROC curve of csPCa using PSAD, BD and BD+PSAD.

We plotted the ROC curve of a positive biopsy using PSAD and the BD (**Figure 4**). The BD (AUC=0.74, 95% CI:0.64-0.85, p<0.05) was associated with PCa detection and, when combined with PSAD (AUC=0.64, 95% CI:0.48-0.80, p<0.05), had a higher predictive value. We plotted the ROC curve of csPCa detection using the BD and PSAD (**Figure 5**), and the BD (AUC=0.80, 95% CI:0.69-0.91, p<0.05) had a high predictive value for csPCa. The biopsy density combined with PSAD increased the prediction of csPCa (AUC=0.90, 95% CI:0.85-0.97, p<0.05). The threshold value of the BD was determined using the Youden index as 0.42 needles per milliliter.

### Discussion

Prostate cancer is the most prevalent malignancy of men in Europe and the United States (8). Prostate biopsy has been the gold standard for the diagnosis of prostate cancer. Transperineal template–guided biopsy can reach the anterior areas of the prostate and can operate more cores to achieve a greater cancer detection rate (9). With the development of imaging techniques and the introduction of PI-RADS, prostate biopsy is now mainly used in targeted biopsy with several needles for PI-RADS 4-5 patients; biopsy was operated as exhaustive as possible for patients with PI-RADS 1-2 but with a highly clinical suspicion of PCa. PIRADS 3 was a “gray zone.” It did not show statistical difference in the cancer detection rate of PCa or csPCa for PI-RADS 3 patients who underwent target biopsy versus those with a systematic biopsy (10). Considering that a PI-RADS 3 lesion was defined as an equivocal lesion, previous research (11) has suggested that PSAD should be combined to decide whether to operate biopsy for PI-RADS 3 patients.

It was hard to distinguish the prostate cancer from other diseases using mpMRI. A total of 115 patients with PI-RADS 1-2 but with a highly clinical suspicion of prostate cancer were included in our study. Approximately 14 patients were PCa, 7 of which were csPCa, and the rest (101 patients) mostly had a pathological diagnosis of lymphocytic infiltration or benign prostatic hyperplasia(BPH). In fact, it had difficulty to differentiate between prostate cancer and prostatitis, since prostatitis can mimic cancer on MRI due to an overlap of signal alterations between these two entities. Both entities could show a decrease of citrate and an increase of choline on magnetic resonance spectroscopy (MRS). Research suggested that the mpMRI ADC sequence ≥900 mm^2^/s was a good indicator to distinguish prostatitis from prostate cancer (12). BPH and prostate cancer were difficult to differentiate and often coexisted. Most cancers arised in prostates with concomitant BPH (83.3%), and cancer was incidentally found in a significant number of transurethral prostatectomy (TURP) specimens (10%) (13). Actually, BPH was rated as 2 in the PI-RADS assessment for T2W for the transition zone (5). It had reported that BPH in large prostates may be protective of PCa and 12–16- core biopsy in larger prostates may be more likely missing the cancer lesion (14). It was proposed that the use of a 5α-reductase inhibitor may improve the CDR of PCa. A short-term pretreatment of prostate surgery with a dual 5α-reductase inhibitor decreased blood loss in a large prostate (15) and resulted in a reduction in the total prostate volume, which could, in theory, enhance prostate cancer detection by reducing the benign component of the gland (16).

We found that the prostate volume was statistically significant in each group of our study. Previous research had reported that the prostate volume was inversely correlated with prostate cancer detection (14, 17). Choosing a biopsy strategy in terms of the prostate volume had been suggested in the prostate biopsy. A biopsy strategy of at least 8 cores was performed for patients with a prostate volume <30 ml, and for patients with a prostate volume >30 ml, at least 12 cores were performed (18). Then, a Vienna nomogram based on the prostate volume and age were constructed to determine biopsy cores for a higher positive biopsy-positive rate (3). However, Leitão et al. (19) compared a Vienna nomogram-guided prostate biopsy with a 10-core systematic biopsy and revealed no significant differences in the cancer detection rate with 42.6% vs. 38.4% (P =0.705). Differed from the cancer detection rate above, an Asian country (20) reported a detection rate of 20.5% using the Vienna nomogram but noted that there was no significant difference from the laterally directed sextant and octant biopsy methods (17.6%).

In the era of target biopsy, it existed as controversial to perform the biopsy in patients with an MRI low-possibility of prostate cancer. The PRECISION study (21) suggested that prostate biopsy can be ignored if mpMRI reports were negative. The PROMIS (22), a prospective, multi-center, paired-cohort, confirmatory study, chose transperineal-guided prostate mapping (TPM) biopsy as a gold standard comparing TRUS biopsy with mp-MRI and concluded that mpMRI could avoid an overdiagnosis of prostate biopsy in a quarter of patients. However, in this study, we could not deny that prostate biopsy showed better specificity and a positive predictive value. In PROMIS, out of 158 patients with an mpMRI diagnosis of non-significant cancer 17 had a TPM diagnosis of csPCa. The mainstream view was that biopsy was not negligible. The European multicenter study MRI-FIRST (23), which included 275 biopsy-naive patients, performed biopsy after mpMRI and concluded that mpMRI combined with systemic biopsy improved the cancer detection rate and MRI could not replace biopsy. A single-center PICTURE study (24) evaluated mpMRI versus biopsy in repeat biopsy patients and concluded that mpMRI missed a portion of csPCa. In addition, novel diagnostic tools could assist the diagnosis of PCa. Artificial intelligence (AI), through the machine learning (ML) and deep learning (DL) model to teach computers to learn by example, was applied to diagnostic imaging. It showed better specificity and sensitivity combined with PI-RADS v2 in the detection of prostate lesions (25). SelectMDx was another biomarker-based risk score tool for PCa, and it had demonstrated superior specificity compared with mpMRI. SelectMDx was advised to perform after an initial negative mpMRI to avoid unnecessary biopsy (26).

PSAD was associated with csPCa, and BD combined with PSAD could increase the csPCa detection rate in our result. In fact, it had been reported that the PSA density was correlated with csPCa detection, and lower PSAD offered the avoidance of prostate biospy in MRI-negative patients in many studies (11, 27, 28). The PSA density was an important indicator for deciding whether to perform prostate biopsy in MRI-negative patients. However, it was unknown how many cores were needed to perform prostate biopsy in patients who were MRI negative but require prostate biopsy based on the results of the PSA density. The Vienna nomogram or prostate volume application in this group of patients was not found to have an advantage. In our study, we introduced the BD, which was originally proposed in TPM biopsy. Mortezavi et al. (29) evaluated 415 men who underwent mpMRI followed by TPM. Of those without a suspicious lesion on MRI, 32 (25.8%) were found to have csPCa on TPM. Nakai et al. (30) first performed the one-needle-per-milliliter biopsy method (BD1) for patients with repeat biopsy and showed a cancer detection rate of 51.5%. Then, Stone et al. (4) first defined the BD. He systematically analyzed biopsy-naïve and repeat-biopsy patients’ data and suggested that a BD of more than 1.5 needles per milliliter increased the csPCa detection rate. Differed from Nakai and Stone, in our study, we focused on a patient with PIRADS <3 and brought the BD to a 14-region transperineal template-guided biopsy (rather than TPM) and found a threshold value of the BD.

We used binary logistics regression to find that the BD was associated with the detection of csPCa. Prostate biopsy can be broadly divided into two strategies. The first was <20 cores of prostate biopsy. Bo-Ren Wang et al. (31) performed 18- and 14-core transperineal biopsy randomly in repeat-biopsy patients and the cancer detection rate was 33.1% vs. 17.6% with an average prostate volume of 48.80 (BD0.37) vs. 45.65 (BD0.31). Ying-Hao Sun et al. (32) divided the prostate into 20 regions and performed one needle per region. In 216 biopsy-naive patients with a cancer detection rate of 35.4%, the mean prostate volume was 39.88 ml, and the BD was 0.5 needles per milliliter. The second was saturation biopsy: >20 cores of prostate biopsy. Taira et al. (33) performed a transperineal template–guided saturation prostate biopsy in 79 biopsy-naïve men (mean 55.1 cores). The biopsy density was 1.2 needles per milliliter and the CDR was 75.9%. Bittner et al. (34) reported a more exhaustive saturation biopsy with TPM in binaïvenaive patients, which divided the prostate into the base and apex layers and operated biopsy at 5-mm intervals using the brachytherapy grid. The cancer detection rate achieved 78.2% with mean needles of 63.1 (BD=1.7). It was evident from the above study that regardless of the biopsy strategy was the <20 core of prostate biopsy or >20 cores; the prostate cancer detection rate increased with the increasing BD. In our research, we further analyzed PI-RADS <3 patients and found that the csPCa detection rate increased with increasing BD. It was concluded that BD of more than 0.42 needles per milliliter had a higher detection rate of csPCa according to the Youden index and BD (AUC=0.70, 95% CI:0.60-0.80, p<0.05) combined with PSAD was a better predictor of csPCa according to the ROC curves (AUC=0.90, 95% CI:0.85-0.97, p<0.05).

There are some shortcomings in this study. First, it is a retrospective study, which is prone to selection bias; second, the amount of csPCa patients is few, more patients and multi-center studies are in progress. Then, in prostate biopsy, the number of cores was probably determined by the biopsy experience of the operator, PSA level, prostate volume, and other factors without an exact standard for the drawback of a retrospective study. We will validate it in our further study.

In conclusion, the BD is related to csPCa. In PI-RADS<3 patients, BD and PSAD are related to csPCa. A biopsy density of more than 0.42 needles per milliliter can increase the csPCa detection rate, which should be considered as an alternative biopsy method when we perform prostate biopsy in patients with PI-RADS<3.

## Data Availability Statement

The raw data supporting the conclusions of this article will be made available by the authors, without undue reservation.

## Ethics Statement 

This original research was approved by the research ethics committee of Northern Jiangsu People’s Hospital (2017KY-015), Yangzhou, Jiangsu, China. The patients/participants provided their written informed consent to participate in this study.

## Author Contributions

Contributions: (I) Conception and design: HZ, X-fD. (II) administrative support: HZ, S-mL. (III) Provision of study materials or patients: HZ, S-yP. (IV) Collection and assembly of data: L-yZ, QX. (V) Data analysis and interpretation: ZL (6th Author), YL. (VI) Manuscript writing: All authors. (VII) Final approval of manuscript: all authors.

## Funding

This work was supported the Jiangsu Provincial Commission of Health and Family Planning Research Project (no. H201550).

## Conflict of Interest

The authors declare that the research was conducted in the absence of any commercial or financial relationships that could be construed as a potential conflict of interest.

## Publisher’s Note

All claims expressed in this article are solely those of the authors and do not necessarily represent those of their affiliated organizations, or those of the publisher, the editors and the reviewers. Any product that may be evaluated in this article, or claim that may be made by its manufacturer, is not guaranteed or endorsed by the publisher.

## References

[1] Johnson DC Raman SS Mirak SA Kwan L Bajgiran AM Hsu W. Detection of Individual Prostate Cancer Foci *via* Multiparametric Magnetic Resonance Imaging. Eur Urol (2019) 75(5):712–20. doi: 10.1016/j.eururo.2018.11.031 30509763

[2] van Houdt PJ Ghobadi G Schoots IG Heijmink SWTPJ de Jong J van derPoel HG. Histopathological Features of MRI-Invisible Regions of Prostate Cancer Lesions. J Magn Reson Imaging (2020) 51(4):1235–46. doi: 10.1002/jmri.26933 31588646

[3] Remzi M Fong YK Dobrovits M Anagnostou T Seitz C Waldert M. The Vienna Nomogram: Validation of a Novel Biopsy Strategy Defining the Optimal Number of Cores Based on Patient Age and Total Prostate Volume. J Urol (2005) 174(4 Pt 1):1256–61. doi: 10.1097/01.ju.0000173924.83392.cc 16145388

[4] Stone NN Crawford ED Skouteris VM Arangua P Metsinis PM Lucia MS. The Ratio of the Number of Biopsy Specimens to Prostate Volume (Biopsy Density) Greater Than 1.5 Improves the Prostate Cancer Detection Rate in Men Undergoing Transperineal Biopsy of the Prostate. J Urol (2019) 202(2):264–71. doi: 10.1097/JU.0000000000000204 30835628

[5] Weinreb JC Barentsz JO Choyke PL Cornud F Haider MA Macura KJ. PI-RADS Prostate Imaging - Reporting and Data System: 2015, Version 2. Eur Urol (2016) 69(1):16–40. doi: 10.1016/j.eururo.2015.08.052 26427566 PMC6467207

[6] Epstein JI Egevad L Amin MB. The 2014 International Society of Urological Pathology (ISUP) Consensus Conference on Gleason Grading of Prostatic Carcinoma: Definition of Grading Patterns and Proposal for a New Grading System. Am J Surg Pathol (2016) 40(2):244–52. doi: 10.1097/PAS.0000000000000530 26492179

[7] Matoso A Epstein JI. Defining Clinically Significant Prostate Cancer on the Basis of Pathological Findings. Histopathol (2019) 74(1):135–45. doi: 10.1111/his.13712 30565298

[8] Siegel RL Miller KD Jemal A. Cancer Statistics, 2020. CA: Cancer J Clin (2020) 70(1):7–30. doi: 10.3322/caac.21590 31912902

[9] Sivaraman A Sanchez-Salas R Barret E Ahallal Y Rozet F Galiano M. Transperineal Template-Guided Mapping Biopsy of the Prostate. Int J Urol (2015) 22(2):146–51. doi: 10.1111/iju.12660 25421717

[10] Maggi M Panebianco V Mosca A Salciccia S Gentilucci A Di Pierro G. Prostate Imaging Reporting and Data System 3 Category Cases at Multiparametric Magnetic Resonance for Prostate Cancer: A Systematic Review and Meta-Analysis. Eur Urol Focus (2020) 6(3):463–78. doi: 10.1016/j.euf.2019.06.014 31279677

[11] Boesen L Nørgaard N Løgager V Balslev I Bisbjerg R Thestrup KC. Prebiopsy Biparametric Magnetic Resonance Imaging Combined With Prostate-Specific Antigen Density in Detecting and Ruling Out Gleason 7-10 Prostate Cancer in Biopsy-Naïve Men. Eur Urol Oncol (2019) 2(3):311–9. doi: 10.1016/j.euo.2018.09.001 31200846

[12] Meier-Schroers M Kukuk G Wolter K Decker G Fischer S Marx C. Differentiation of Prostatitis and Prostate Cancer Using the Prostate Imaging-Reporting and Data System (PI-RADS). Eur J Radiol (2016) 85(7):1304–11. doi: 10.1016/j.ejrad.2016.04.014 27235878

[13] Bostwick DG Cooner WH Denis L Jones GW Scardino PT Murphy GP. The Association of Benign Prostatic Hyperplasia and Cancer of the Prostate. Cancer (1992) 70(1 Suppl):291–301. doi: 10.1002/1097-0142(19920701)70:1+<291::aid-cncr2820701317>3.0.co;2-4 1376199

[14] Al-Khalil S Boothe D Durdin T Sunkara S Watkins P Yang S. Interactions Between Benign Prostatic Hyperplasia (BPH) and Prostate Cancer in Large Prostates: A Retrospective Data Review. Int Urol Nephrol (2016) 48(1):91–7. doi: 10.1007/s11255-015-1146-2 26590832

[15] Busetto GM Giovannone R Antonini G Rossi A Del Giudice F Tricarico S. Short-Term Pretreatment With a Dual 5α-Reductase Inhibitor Before Bipolar Transurethral Resection of the Prostate (B-TURP): Evaluation of Prostate Vascularity and Decreased Surgical Blood Loss in Large Prostates. BJU Int (2015) 116(1):117–23. doi: 10.1111/bju.12917 25291499

[16] Serfling R Shulman M Thompson GL Xiao Z Benaim E Roehrborn CG. Quantifying the Impact of Prostate Volumes, Number of Biopsy Cores and 5alpha-Reductase Inhibitor Therapy on the Probability of Prostate Cancer Detection Using Mathematical Modeling. J Urol (2007) 177(6):2352–6. doi: 10.1016/j.juro.2007.01.116 17509357

[17] Chen ME Troncoso P Johnston D ang K Babaian RJ. Prostate Cancer Detection: Relationship to Prostate Size. Urology (1999) 53:764–8.doi: 10.1016/s0090-4295(98)00574-3 10197853

[18] Ficarra V Novella G Novara G Galfano A Pea M Martignoni G. The Potential Impact of Prostate Volume in the Planning of Optimal Number of Cores in the Systematic Transperineal Prostate Biopsy. Eur Urol (2005) 48(6):932–7. doi: 10.1016/j.eururo.2005.08.008 16202510

[19] Leitão TP Alfarelos J Rodrigues T Pereira Silva E R Garcia RM Martinho D. A Prospective Randomized Trial Comparing the Vienna Nomogram and a Ten-Core Prostate Biopsy Protocol: Effect on Cancer Detection Rate. Clin Genitourin Cancer (2017) 15(1):117–21. doi: 10.1016/j.clgc .2016.06.003 10.1016/j.clgc.2016.06.003 27436153

[20] Singam P Bahadzor B Abas A Hee TG Ho C Hong GE. Prostate Cancer Detection via Transrectal Ultrasound Biopsy: Vienna Nomogram Versus Sextant/Octant Biopsy Methods. Urotoday Int J (2012) 5:art 47. doi: 10.3834/uij.1944-5784.2012.10.06

[21] Merrett C Mannas M Black PC Zargar H. Magnet Before the Needle Commentary on: MRI-Targeted or Standard Biopsy for Prostate-Cancer Diagnosis (PRECISION Trial). Urology (2018) 118:1–2. doi: 10.1016/j.urology.2018.04.024 29730255

[22] Ahmed HU El-Shater Bosaily A Brown LC Gabe R Kaplan R Parmar MK. Diagnostic Accuracy of Multi-Parametric MRI and TRUS Biopsy in Prostate Cancer (PROMIS): A Paired Validating Confirmatory Study. Lancet (2017) 389(10071):815–22. doi: 10.1016/S0140-6736(16)32401-1 28110982

[23] Rouvière O Puech P Renard-Penna R Claudon M Roy C Mège-Lechevallier F. Use of Prostate Systematic and Targeted Biopsy on the Basis of Multiparametric MRI in Biopsy-Naive Patients (MRI-FIRST): A Prospective, Multicentre, Paired Diagnostic Study. Lancet Oncol (2019) 20(1):100–9. doi: 10.1016/S1470-2045(18)30569-2 30470502

[24] Simmons LAM Kanthabalan A Arya M Briggs T Barratt D Charman SC. The PICTURE Study: Diagnostic Accuracy of Multiparametric MRI in Men Requiring a Repeat Prostate Biopsy. Br J Cancer (2017) 116(9):1159–65. doi: 10.1038/bjc.2017.57 PMC5418442 28350785

[25] Tătaru OS Vartolomei MD Rassweiler JJ Virgil O Lucarelli G Porpiglia F. Artificial Intelligence and Machine Learning in Prostate Cancer Patient Management-Current Trends and Future Perspectives. Diagnostics (Basel) (2021) 11(2):354. doi: 10.3390/diagnostics11020354 33672608 PMC7924061

[26] Maggi M Del Giudice F Falagario UG Cocci A Russo GI Di Mauro M. SelectMDx and Multiparametric Magnetic Resonance Imaging of the Prostate for Men Undergoing Primary Prostate Biopsy: A Prospective Assessment in a Multi-Institutional Study. Cancers (Basel) (2021) 13(9):2047. doi: 10.3390/cancers13092047 33922626 PMC8122883

[27] Yang S Zhao W Tan S Zhang Y Wei C Chen T. Combining Clinical and MRI Data to Manage PI-RADS 3 Lesions and Reduce Excessive Biopsy. Transl Androl Urol (2020) 9(3):1252–61. doi: 10.21037/tau-19-755 PMC7354292 32676408

[28] Anastay V Gondran-Tellier B McManus R Delonca R Akiki A Gaillet S. Nonsuspicious Prebiopsy Multiparametric MRI: Is Prostate Biopsy Still Necessary? Abdom Radiol (NY) (2020) 45(12):4160–5. doi: 10.1007/s00261-020-02728-8 32902661

[29] Mortezavi A Marzendorfer O Donati OF Rizzi G Rupp NJ Wettstein MS. Diagnostic Accuracy of Multiparametric Magnetic Resonance Imaging and Fusion Guided Targeted Biopsy Evaluated by Transperineal Template Saturation Prostate Biopsy for the Detection and Characterization of Prostate Cancer. J Urol (2018) 200:309. doi: 10.1016/j.juro.2018.02.067 29474846

[30] Nakai Y Tanaka N Anai S Miyake M Hori S Tatsumi Y. Transperineal Template-Guided Saturation Biopsy Aimed at Sampling One Core for Each Milliliter of Prostate Volume: 103 Cases Requiring Repeat Prostate Biopsy. BMC Urol (2017) 17(1):28. doi: 10.1186/s12894-017-0219-1 28381267 PMC5382378

[31] Wang BR Chen CC Zheng RH Hu JC Ou YC. Comparison of Cancer Detection Between 18- and 12-Core Prostate Biopsy in Asian Patients With Prostate-Specific Antigen Levels of 4-20 Ng/Ml. J Chin Med Assoc (2018) 81(12):1044–51. doi: 10.1016/j.jcma.2018.06.003 30100355

[32] He BM Chen R Shi ZK Xiao GA Li HS Lin HZ. Trans-Perineal Template-Guided Mapping Biopsy vs. Freehand Trans-Perineal Biopsy in Chinese Patients With PSA < 20 Ng/Ml: Similar Cancer Detection Rate But Different Lesion Detection Rate. Front Oncol (2019) 9:758. doi: 10.3389/fonc.2019.00758 31448239 PMC6696794

[33] Taira AV Merrick GS Galbreath RW Andreini H Taubenslag W Curtis R. Performance of Transperineal Template-Guided Mapping Biopsy in Detecting Prostate Cancer in the Initial and Repeat Biopsy Setting. Prostate Cancer Prostatic Dis (2010) 13(1):71–7. doi: 10.1038/pcan.2009.42 PMC2834351 19786982

[34] Bittner N Merrick GS Bennett A Butler WM Andreini HJ Taubenslag W. Diagnostic Performance of Initial Transperineal Template-Guided Mapping Biopsy of the Prostate Gland. Am J Clin Oncol (2015) 38(3):300–3. doi: 10.1097/COC.0b013e31829a2954 23764680

